# Supplementary material for: Heavy Metal Health Risk Assessment in Picea abies L. Forests Along an Altitudinal Gradient in Southern Romania
Source: Plants (Basel). 2025 Mar 19;14(6):968. doi: 10.3390/plants14060968 (PMC11944697; doi:10.3390/plants14060968)
Supplement: Supplementary file 1 [file plants-14-00968-s001.zip › plants-3508811-supplementary.pdf]

Low litter-plant transfer and extreme bioconcentration factor in needles reflect imbalanced minerals from air pollution under altitudinal gradient in a natural park in South Romania

Constantin Nechita [1,*], Andreea Maria Iordache [2,*], Carmen Roba [3], Claudia Sandru [2], Ramona Zgavarogea [2], and J. Julio Camarero [4]

[1] Department of Biometry, National Institute for Research and Development in Forestry "Marin Drăcea", Calea Bucovinei, 73 bis, 725100, Câmpulung Moldovenesc, Romania; constantin.nechita@icas.ro

[2] National Research and Development Institute for Cryogenics and Isotopic Technologies – ICSI Rm. Valcea, 4 Uzinei Street, 240050 Rm. Valcea, Valcea, Romania; andreea.iordache@icsi.ro; claudia.sandru@icsi.ro; ramona.zgavarogea@icsi.ro

[3] Faculty of Environmental Science and Engineering, Babes-Bolyai University, 30 Fântânele Street, 400294 Cluj-Napoca, Romania; carmen.roba@ubbcluj.ro

[4] Instituto Pirenaico de Ecología (IPE-CSIC), Avda. Montañana 1005, 50080 Zaragoza, Spain; jjcamarero@ipe.csic.es

* Correspondence: andreea.iordache@icsi.ro; constantin.nechita@icas.ro

Supplementary material

Table S1. Criteria for interpreting health risks indices.

Indices	Interval of values	Interpretation
BCF	≤ 1	plants only absorb metals
	> 1	plants have the potential for accumulation
I_{geo}	≤ 1	uncontaminated
	$0 \leq I_{geo} < 1$	uncontaminated to moderately contaminated
	$1 \leq I_{geo} < 2$	moderately contaminated
	$2 \leq I_{geo} < 3$	moderately to heavily contaminated
	$3 \leq I_{geo} < 4$	heavily contaminated
	$4 \leq I_{geo} < 5$	heavily to extremely contaminated
	$I_{geo} \geq 5$	extremely contaminated
C_f	$C_f < 1$	low contamination
	$1 < C_f < 3$	moderate contamination
	$3 < C_f < 6$	considerable contamination
	$C_f > 6$	very high contamination
PLI	PLI < 1	uncontaminated
	1 ≤ PLI <2	uncontaminated to moderately contaminated
	2 ≤ PLI <3	moderately to strongly contaminated
	PLI ≥ 3	strongly contaminated
$PI_{Nemerow}$	≤ 0.7	uncontaminated
	0.7-1	danger range
	1-2	low contamination
	2-3	moderate contamination
	≥ 3	severe contamination
E_r^i	$E_r^i < 40$	low
	$40 \leq E_r^i < 80$	moderate
	$80 \leq E_r^i < 160$	considerable
	$160 \leq E_r^i < 320$	high
	$320 \geq E_r^i$	very high
PERI	RI < 150	moderate
	150 ≤ RI < 300	considerable
	300 ≤ RI < 600	very high
	600 ≥ RI	

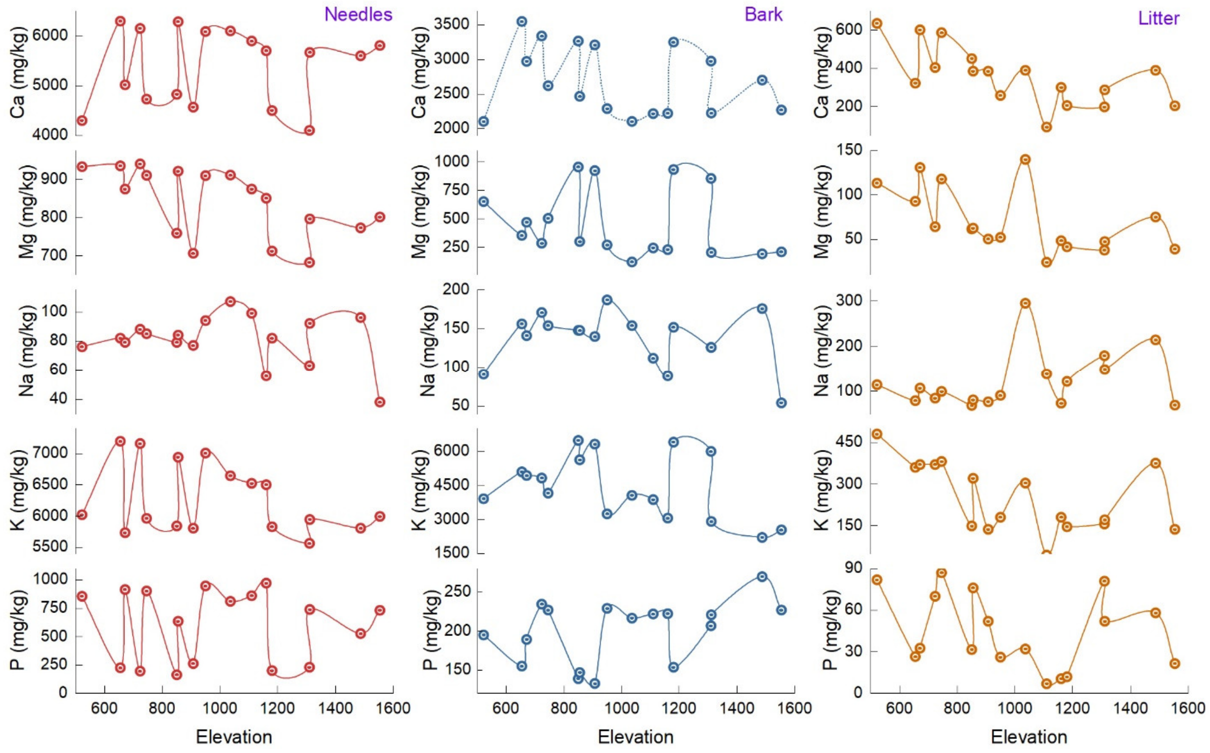

Figure S1. The trends of nutrients in relationship with altitude.

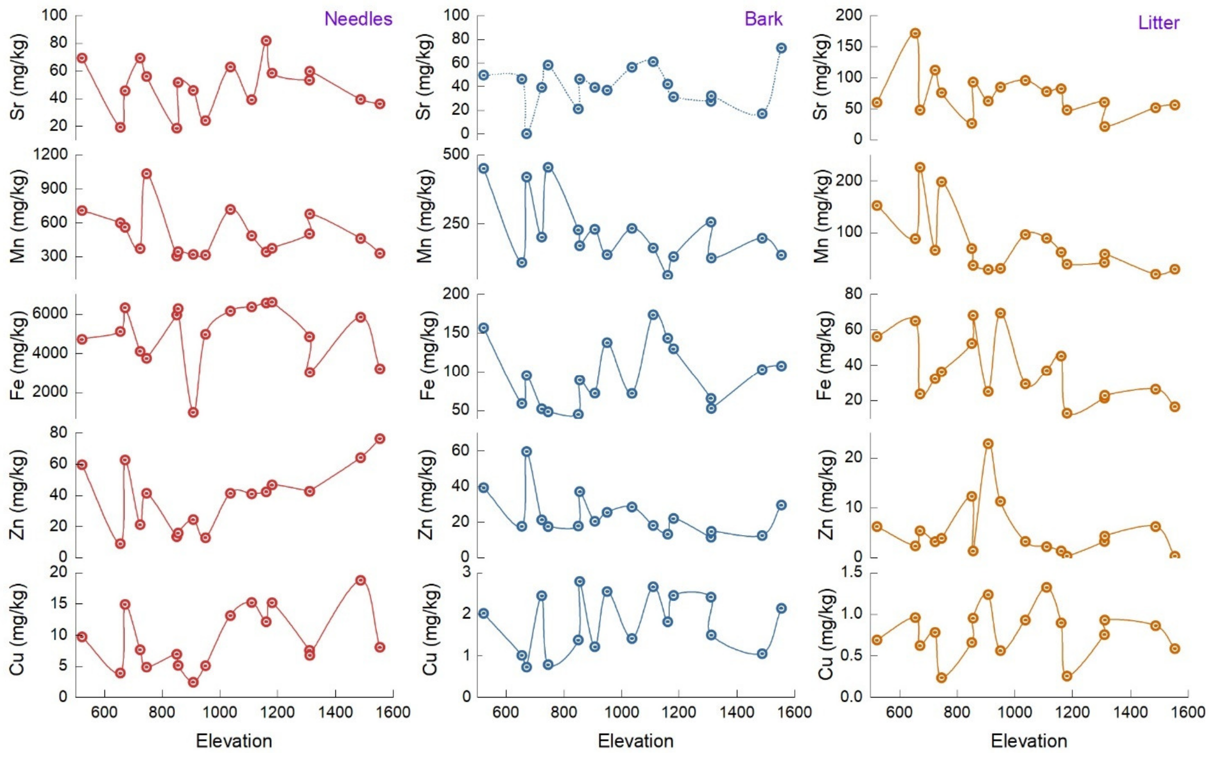

Figure S2. The trends of non-toxic metals in relationship with altitude.

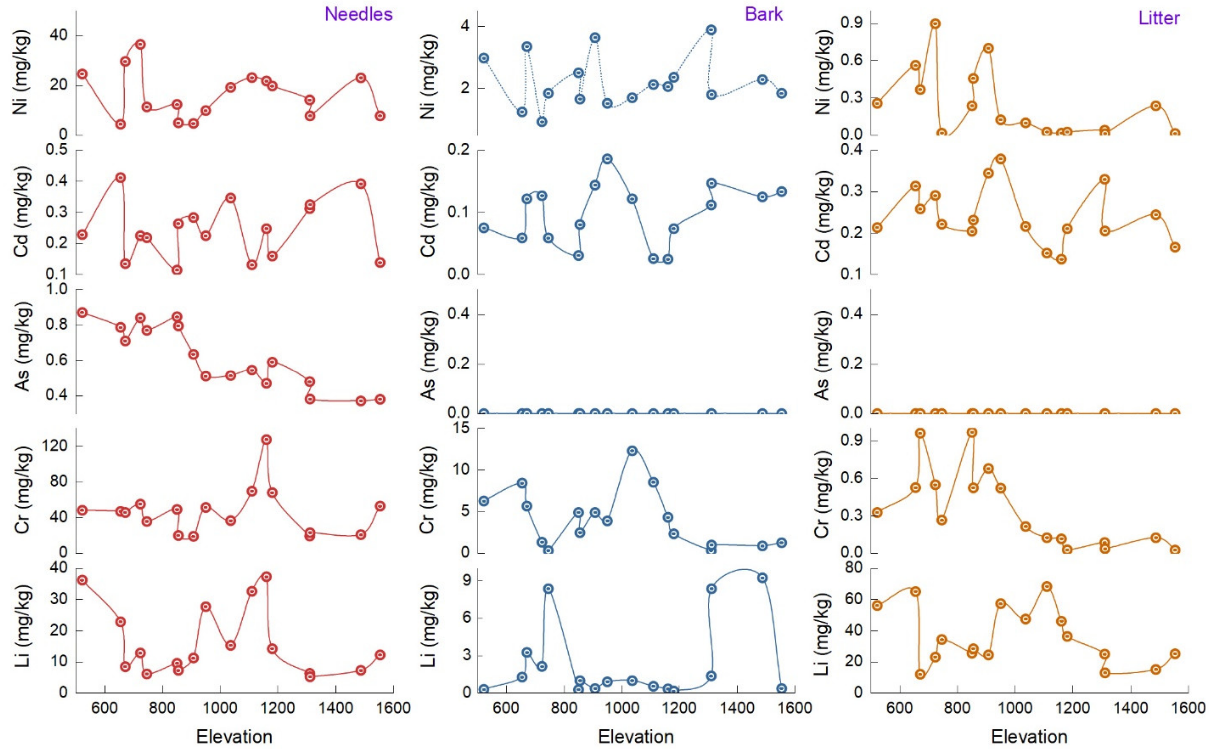

Figure S3. The trends of toxic metals in relationship with altitude.
